# Effects of Composition of Iron-Cross-Linked Alginate Hydrogels for Cultivation of Human Dermal Fibroblasts

**DOI:** 10.1155/2012/820513

**Published:** 2012-12-13

**Authors:** Ikuko Machida-Sano, Sakito Ogawa, Hiroyuki Ueda, Yoshitaka Kimura, Nao Satoh, Hideo Namiki

**Affiliations:** Department of Biology, School of Education, Waseda University, 2-2 Wakamatsu-cho, Shinjuku-ku, Tokyo 162-8480, Japan

## Abstract

We investigated the suitability of ferric-ion-cross-linked alginates (Fe-alginate) with various proportions of L-guluronic acid (G) and D-mannuronic acid (M) residues as a culture substrate for human dermal fibroblasts. High-G and high-M Fe-alginate gels showed comparable efficacy in promoting initial cell adhesion and similar protein adsorption capacities, but superior cell proliferation was observed on high-G than on high-M Fe-alginate as culture time progressed. During immersion in culture medium, high-G Fe-alginate showed little change in gel properties in terms of swelling and polymer content, but the properties of high-M Fe-alginate gel were altered due to loss of ion cross-linking. However, the degree of cell proliferation on high-M Fe-alginate gel was improved after it had been stabilized by immersion in culture medium until no further changes occurred. These results suggest that the mode of cross-linkage between ferric ions and alginate differs depending on alginate composition and that the major factor giving rise to differences in cell growth on the two types of Fe-alginate films is gel stability during culture, rather than swelling of the original gel, polymer content, or protein adsorption ability. Our findings may be useful for extending the application of Fe-alginate to diverse biomedical fields.

## 1. Introduction

In tissue engineering research, it is necessary to develop effective culture substrates for cells to facilitate regeneration of deficient tissue. It has been shown that the environment surrounding cells has a significant effect on cell behavior. The physical conditions of any scaffold to which cells adhere are an important determinant of cell growth [1–6], along with biochemical signals. Cells receive various types of information from the culture substrate, such as surface stiffness [1–4] or roughness [5, 6]. Therefore, a good understanding of the relationship between cellular behavior and the physical characteristics of culture substrates is critical for development of effective scaffolds that allow suitable cell adhesion, proliferation, and differentiation.

In a previous study, we demonstrated that ferric-ion-cross-linked alginate (Fe-alginate) was an efficient material for use as scaffold, allowing good cell adhesion and proliferation [7]. Alginates are a material widely used in various biomedical fields, and are composed of 1,4-linked *β*-D-mannuronic acid (M) and *α*-L-guluronic acid (G) residues, forming gels with certain multivalent metal ions [8, 9]. Calcium ions are used most frequently as a cross-linking agent for alginates, but calcium-ion-cross-linked alginate (Ca-alginate) is known to be an unfavorable culture substrate for cells. Fe-alginate is able to overcome the deficiencies of Ca-alginate, such as poor protein adsorptive capacity, and has been established as an effective cell culture substrate.

Alginates are commonly derived from seaweed, and have widely varying proportions and sequences of two the M and G monomers depending on the source from which they are derived [8, 10]. It is known that the M/G ratio influences the mechanical properties of Ca-alginate gels: those with a high G-content have higher mechanical strength than those with a high M-content [8]. It has also been reported that high-G Ca-alginate forms more porous gels, and that the cross-linkages tend to be more stable over a longer period, than is the case for high-M alginate [11].

In the case of Fe-alginate, it is considered that the alginate composition can also influence the condition of the gel. However, the effects of Fe-alginate substrate composition on gel properties and the growth of cultured cells have not been clarified. The aim of this study was to define the relationship between Fe-alginate composition and the growth of cells cultured on Fe-alginate films. We evaluated the efficiency of cell adhesion and proliferation on Fe-alginate films with differing M/G ratios, using normal human dermal fibroblasts (NHDF) as model cells. We also focused on the degree of change in the condition of the Fe-alginate gel during cell culture. Here we report that favorable cell proliferation was observed on high-G, rather than on high-M Fe-alginate gels, and that the stability of Fe-alginate gel with firm cross-linkage between alginate and ferric ions seems to be a key supportive element for good cell proliferation.

## 2. Materials and Methods

### 2.1. Materials

Eagle's minimum essential medium (E-MEM) was obtained from Nissui Pharmaceutical (Tokyo, Japan), fetal bovine serum (FBS) was from Tissue Culture Biologicals (Tulare, CA, USA), and trypsin was from Becton Dickinson (Sparks, MD, USA). NHDF were obtained from Cambrex Bio Science Walkersville, Inc. (Walkersville, MD, USA), and used between the 10th–20th passages. Anti-vitronectin polyclonal antibody was purchased from AbD Serotec (Oxford, UK). Horseradish peroxidase (HRP)-conjugated anti-rabbit IgG was from Santa Cruz Biotechnology (Santa Cruz, CA, USA). All other chemicals were purchased from Wako Pure Chemical Co. (Tokyo, Japan).

### 2.2. Preparation of Alginate Films

Two types of alginate were used in this study: a high G-content alginate (M : G ratio 34 : 66) and a high M-content alginate (M : G ratio 57 : 43). The viscosity (1% (w/v) solution at 20°C) of both alginates, purchased from KIMICA Corporation (Tokyo, Japan), was about 100–200 mPa·s. The procedures for preparation of the Fe-alginate gel films have been described previously [10]. Briefly, 3 mL of 1% (w/v) alginate solution was added to the wells of 6-well tissue culture plates along with cellulosic sheets (BEMCOT, Ozu Corporation, Tokyo, Japan) as frames, and then dried at 60°C overnight. After drying, 3 mL of 500 mM or 50 mM FeCl_3_ was added to each well to induce cross-linkage of the alginate at 25°C for 30 min. The resulting gel films were washed with deionized water to remove unreacted ions on the surface, and sterilized with 70% (v/v) ethanol. Before seeding of cells on the films, the films were stabilized by immersion in E-MEM at 37°C under a 5% CO_2_ atmosphere for 72 h, and the medium was replaced every 24 h.

### 2.3. Cell Culture

NHDF were cultured in E-MEM with 10% (v/v) FBS at 37°C under a 5% CO_2_ atmosphere. To detach cells by trypsinization, the cells were incubated with 0.25% (w/v) trypsin and 0.02% (w/v) EDTA in Ca^2+^-, Mg^2+^-free phosphate-buffered saline (PBS) at 37°C for 10 min. E-MEM containing 10% (v/v) FBS was subsequently added to terminate the enzyme reaction. The cell suspension was centrifuged at 1000 ×*g* for 5 min and resuspended in E-MEM containing 10% (v/v) FBS. The cells were counted with a Coulter Counter (Beckman Coulter Corporation, FL, USA) and seeded onto Fe-alginate films in 6-well tissue culture plates at a density of 4000 cells/cm^2^ in 3 mL of culture medium. Samples were incubated at 37°C under a 5% CO_2_ atmosphere and the medium was replaced every day. After 1, 5, and 8 days, the cells were observed using a phase-contrast microscope and photographed, and then cell counting was performed to quantify the degree of cell proliferation. To count the attached cells, each film was transferred to another plate, and washed with PBS. The cells were detached by trypsinization at 37°C for 10 min, and then resuspended in E-MEM containing 10% (v/v) FBS. The numbers of cells in the suspensions were counted with a Coulter Counter. Cells were also cultured on high M-content Fe-alginate films after immersion in E-MEM at 37°C under a 5% CO_2_ atmosphere for 8 days to stabilize the gel condition.

### 2.4. Protein Adsorption on the Alginate Films

Alginate films were immersed in FBS (0.2 mL/cm^2^) in a 6-well tissue culture plate at 37°C for 2 h. After incubation, the films were washed with deionized water, and transferred to new tissue culture plates. Then 1% Triton X-100 (0.1 mL/cm^2^) was added to desorb the proteins on the film surfaces at 37°C for 2 h. Protein concentrations of the desorption fraction were determined using a bicinchoninic acid (BCA) protein assay reagent kit (Thermo Fisher Scientific Pierce Biotechnology, IL, USA) in accordance with the manufacturer's instructions. The solutions were also collected to separate the proteins in the solutions using SDS-PAGE on 10% polyacrylamide gel. An equivalent volume of each sample was applied. Protein bands in the gel were transferred to Immobilon membranes (Millipore, Bedford, MA, USA) for immunoblot analysis. The blotted membranes were treated with 5% (w/v) non-fat dried milk in PBS for blocking, and then incubated with anti-vitronectin antibody diluted with the blocking solution at room temperature for 60 min. After washing with 0.05% (v/v) Tween 20 in PBS, the membranes were incubated with HRP-conjugated secondary antibodies at room temperature for 60 min, and the immunospecific bands on the membranes were visualized by the DAB color reaction method. Quantitative analysis of adsorbed vitronectin was performed using Image J 1.40 g (http://rsb.info.nih.gov/ij).

### 2.5. Assessment of the State of Alginate Films

Alteration of alginate gel conditions during the culture period was evaluated by measuring the degree of swelling and polymer content of the gels. The assessments were conducted at 1, 5, 8, and 16 days after the start of incubation. To investigate the degree of gel swelling, the thickness of the alginate films was determined using a dial thickness gauge (G-7C; Ozaki Mfg. Co., Ltd.). For estimation of polymer content, dry weight was measured after cultivation by weighing the gel that had been dried at 60°C for 2 h.

### 2.6. Statistics

Statistical analysis was performed using KaleidaGraph version 4.0 software. The data were subjected to analysis of variance (ANOVA). Scheffe test was used for post hoc evaluation of differences between the respective groups. For all statistical evaluations, significance was assigned at *P* < 0.05.

## 3. Results

### 3.1. Cell Growth on the Fe-Alginate Films

**Figure 1:**
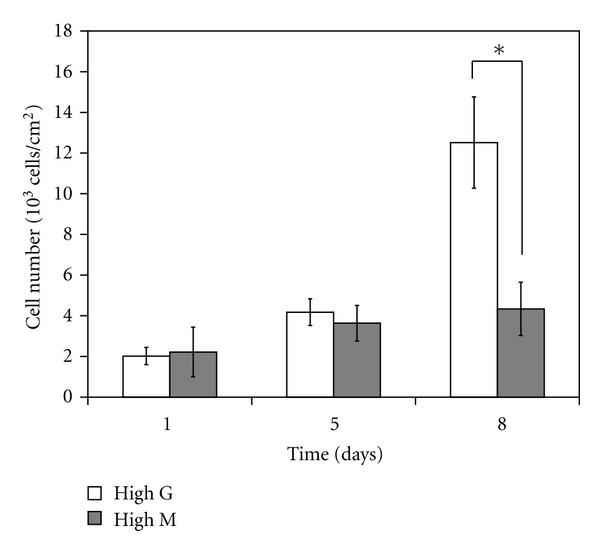
Proliferation of NHDF on Fe-alginate films with a high G or high M content. Cells were seeded on Fe-alginate films at a density of 4000 cells/cm^2^, and numbers of attached cells were counted at 1, 5, and 8 days after the start of culture. Error bars indicate standard deviation for *n* = 5. *P < 0.05.

**Figure 2:**
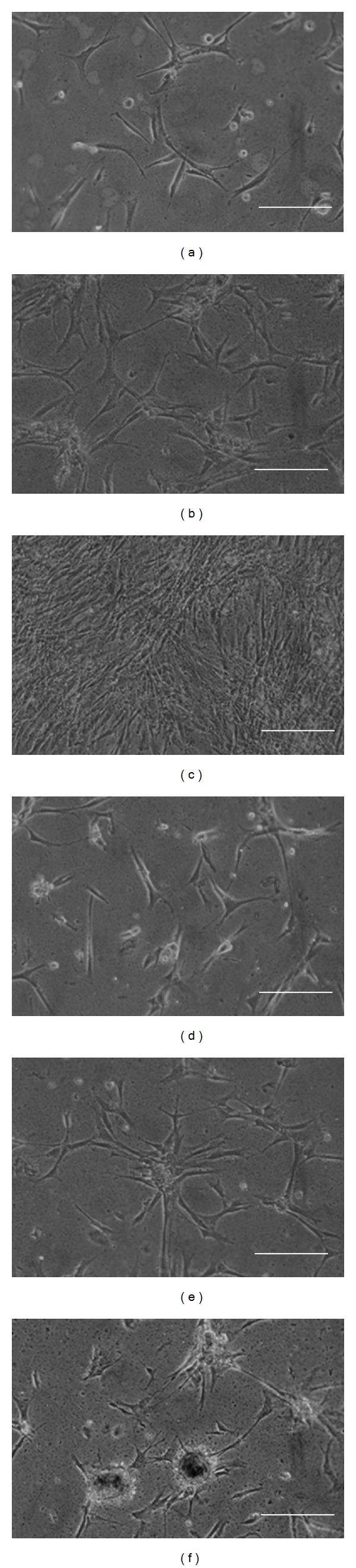
Morphology of cells seeded on Fe-alginate films. Cells were cultured on high-G (a–c) and high-M (d–f) alginate films. Phase-contrast micrographs were taken at 1 day (a, d), 5 days (b, e), and 8 days (c, f) after cell seeding. Bar equals 200 *μ*m.

The adequacy of high G- and M-content Fe-alginate films as culture substrates was investigated by cultivation of NHDF on the respective gels. In the initial stage of culture, the number of attached cells and cell morphology showed little difference between the two Fe-alginates (Figures 1 and 2). However, as culture time progressed, the number of attached cells on the high-G Fe-alginate gel was notably higher than that on the high-M gels (Figure 1). Cells on the high-G Fe-alginate film showed good spread morphology throughout the culture period (Figures 2(a)–2(c)). Conversely, on the high-G Fe-alginate film, many cells became detached from the substrate, while maintaining cell-cell association, after 8 days of culture (Figure 2(f)).

### 3.2. Protein Adsorption Ability of Fe-Alginate Gels

**Figure 3:**
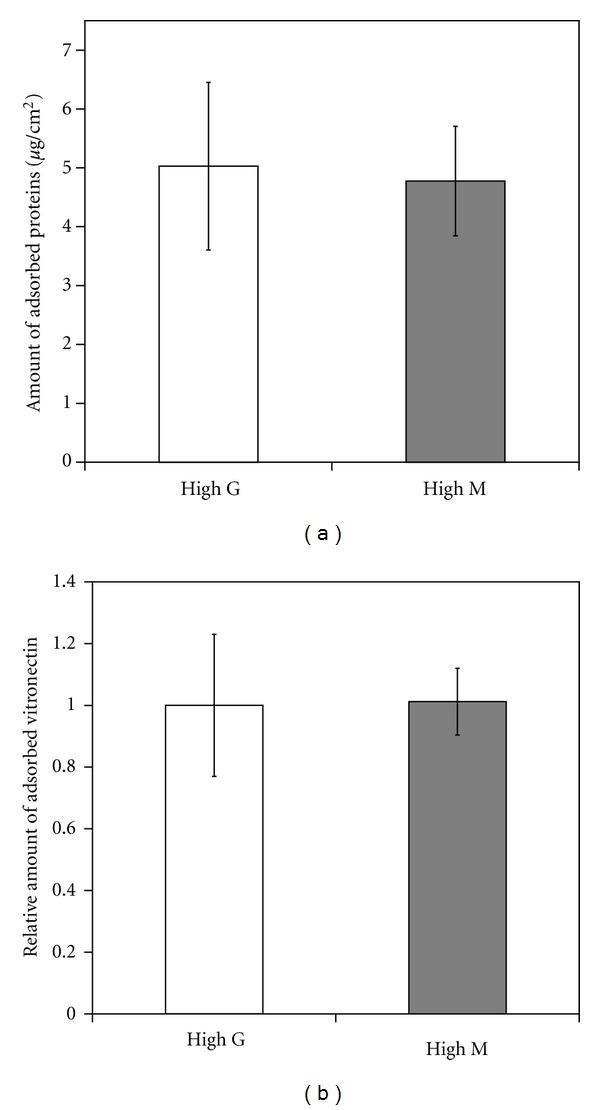
Protein adsorption on Fe-alginate films. The amount of total adsorbed serum proteins was quantified using a BCA protein assay kit (a). Adsorbed serum vitronectin was evaluated by immunoblotting (b). The relative amount of adsorbed vitronectin was normalized relative to that adsorbed on high-G Fe-alginate. Error bars indicate standard deviation for *n* = 3.

The protein adsorption ability of high-G and high-M alginate was investigated. Quantitative analysis demonstrated that both types of gel had an equivalent ability to adsorb serum proteins (Figure 3(a)). Moreover, immunoblot analysis revealed the same degree of adsorption of vitronectin, the major cellular adhesion-related serum protein, on both alginate gels (Figure 3(b)).

### 3.3. States of Fe-Alginate Gels under Physiological Conditions

**Figure 4:**
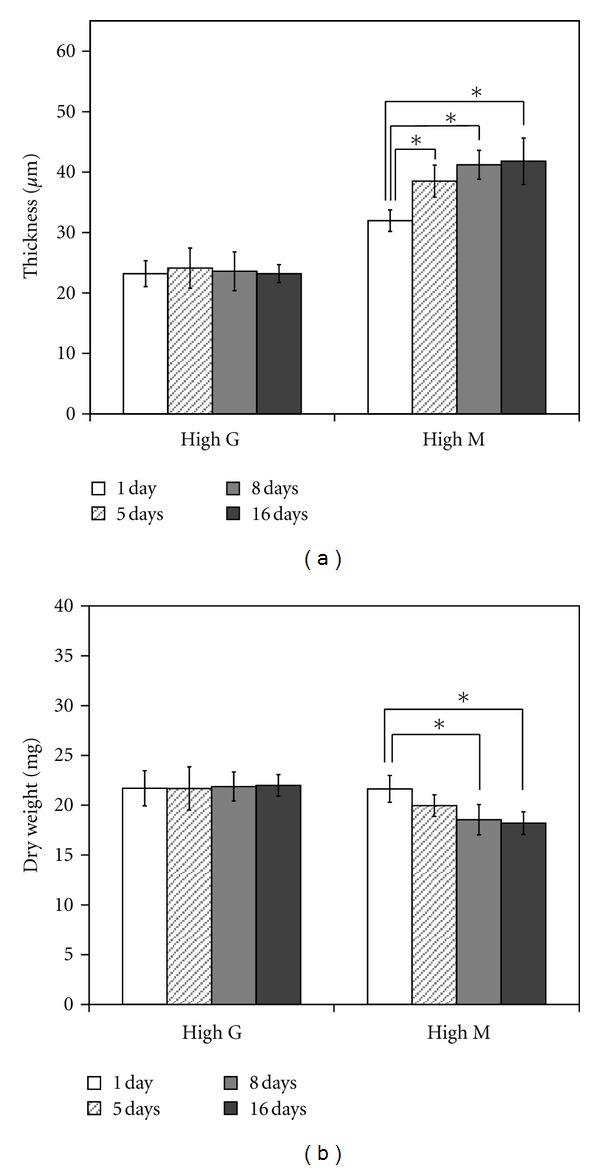
Alterations in Fe-alginate gel properties during culture. Changes in gel properties were determined by measurement of gel thickness (a) and dry weight (b). Error bars indicate standard deviation for *n* = 5. *P < 0.05.

Changes in the conditions of Fe-alginate gel in the culture medium were evaluated by measurement of gel thickness and dry weight (Figure 4). Gel thickness gave an indication of the degree of swelling, whereas dry weight reflected the degree of gel disintegration. The conditions of the high-G Fe-alginate gel showed no significant change even after 16 days. In contrast, the high-M Fe-alginate gel became thicker, and the dry weight decreased with time. The rate of change in the properties of the high-M Fe-alginate gel was rapid until around 8 days, but became more stable thereafter.

### 3.4. Cell Growth on High-M Alginate Films under Stable Gel Conditions

**Figure 5:**
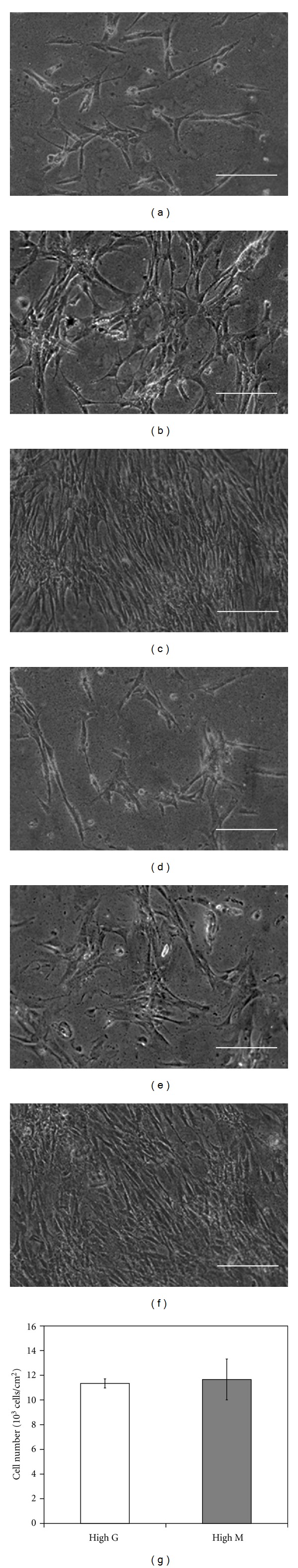
Proliferation of NHDF on Fe-alginate film under stable gel conditions. Cells were seeded onto high-G (a–c) and high-M (d–f) alginate films that had been immersed in culture medium for 8 days to stabilize the gel properties. Phase-contrast micrographs were taken at 1 day (a, d), 5 days (b, e), and 8 days (c, f) after cell seeding. Bar equals 200 *μ*m. The numbers of attached cells on the films were counted after incubation for 8 days (g).

NHDF growth was also observed on Fe-alginate film under stable conditions. Fe-alginate gel was stabilized by immersion in culture medium for 8 days to prevent any further changes in gel condition. Good cell proliferation was confirmed on stable high-M Fe-alginate films at the same level as that on high-G Fe-alginate films (Figure 5).

### 3.5. Effect of the Amount of Cross-Linked Ferric Ions in Fe-Alginates for Support of Cell Growth

**Figure 6:**
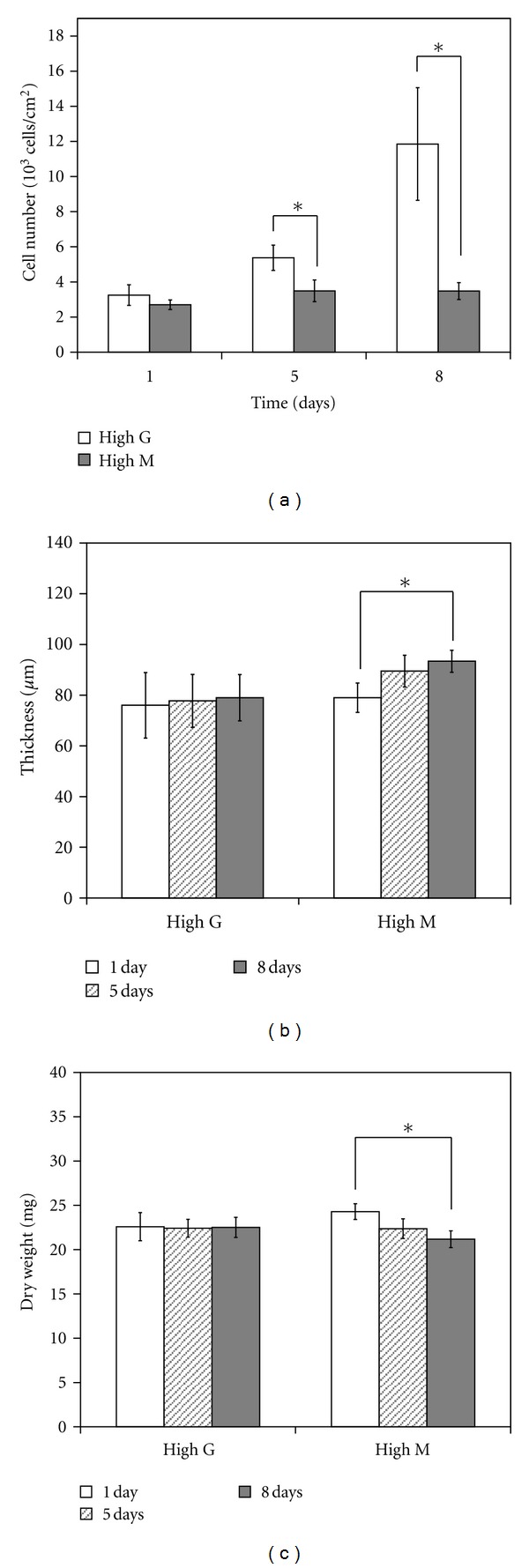
Influence of concentration of ferric ions cross-linked with alginate on the growth of NHDF. Cells were seeded on Fe-alginate gels with a low concentration of ferric ions, being cross-linked with 50 mM FeCl_3_ at a density of 4000 cells/cm^2^, and numbers of attached cells were counted at 1, 5, and 8 days after the start of culture (a). Thickness (b) and dry weight (c) were also measured to evaluate the properties of the Fe-alginate gel. Error bars indicate standard deviation for *n* = 4. *P < 0.05.

To investigate whether the amount of cross-linked ferric ions has a decisive influence on the properties of the two types of alginate, we studied cell growth on Fe-alginate films with a low concentration of cross-linked ions. The concentration of ferric ions for cross-linkage of alginate employed under ordinary circumstances (500 mM FeCl_3_) was determined previously to obtain sufficient film strength and to avoid excessive inhibition of cell growth [7], and the minimum required concentration of FeCl_3_ solution to maintain the film strength needed for cell culture using our methodology was 50 mM. In both types of alginate, cell growth on gels with a low concentration of cross-linked ferric ions was comparable to that on gels with a normal iron concentration (Figure 6(a)). The same tendency was revealed with regard to gel condition (Figures 6(b) and 6(c)), although the thickness of both types of alginate gel increased as the concentration of cross-linked ferric ions decreased (Figure 6(b)).

## 4. Discussions

To establish Fe-alginate as an effective material for biomedical applications in research and therapeutics, an understanding of how the composition of Fe-alginate influences its suitability as a culture substrate for encouraging good cell growth is of considerable significance. In this study, we observed that strong cross-linkage with ferric ions was necessary to ensure good cell proliferation during culture, and that alginate with a high G rather than a high M content showed better conservation of ferric ion cross-linkages.

In the case of Ca-alginate gels, it is reported that the M/G ratio influences cell compatibility. Many researchers have investigated the affinity of Ca-alginate microcapsules and cells. For instance, murine insulinoma cells encapsulated in high-M rather than high-G Ca-alginate showed rapid growth and good metabolic activities [12]. Conversely, in the case of encapsulated neural stem cells, high-G Ca-alginate microcapsules allowed better release of neurotrophic factor [13].

Studies of Ca-alginate as a scaffold for cells have revealed that a high G content promptes good cell growth [14, 15]. Our present results demonstrated superior NHDF cell growth on high-G rather than high-M Fe-alginate films (Figure 1). Additionally, we demonstrated that good cell growth occurred on high-M Fe-alginate films under conditions that tended not to result in gel swelling and disintegration (Figure 5).

It is known that cells recognize proteins that are adsorbed onto the surfaces of substrate materials [16–18]. In particular, vitronectin is a major factor that mediates initial adhesion of NHDF onto Fe-alginate films in the presence of serum [7]. In the present study, there was little difference in the ability to adsorb serum proteins, including vitronectin, between the two types of Fe-alginate gel (Figure 3). High-M and high-G alginate gels showed comparable efficacy to promote initial cell adhesion (Figures 1 and 2), being consistent with their similar protein adsorption capacities.

The interaction between cells and high-M Fe-alginate appeared to be weakened in response to alterations in the condition of the gel. A reduced degree of cell-substratum interaction probably caused cell detachment, although cell-cell interaction was maintained. Swelling and dissolution of high-M Fe-alginate observed in this study may have been caused by loss of cross-linked ferric ions from the gels. Under physiological conditions, disengagement of cross-linking ions from alginate gels occurs in the presence of chelators, monovalent ions, and non-cross-linking divalent ions [19, 20]. It is also reported that high-M Ca-alginate shows loss of mechanical strength during culture [15].

Calcium ions are known to bind to G- and MG-blocks of alginate [21]. However, since interactions between calcium ions and the hydroxyl groups of MG are prevented by the intrinsic configurational characteristics of the MG building blocks, MG sequences in Ca-alginate have a more flexible structure than G sequences [22]. Therefore the structures formed by MG junctions are likely to disassociate in response to the presence of competing ions or application of stress [23]. Although the blocks of alginate involved in binding to ferric ions are unknown, the present study suggested that the binding of ferric ions to alginate might occur through a mechanism similar to that for calcium ions. It can be inferred that ferric ions combine with not only G-blocks but also MG- and/or M-blocks in alginate, because gel swelling and breakage were observed in high-M rather than high-G alginate under physiological conditions (Figure 4). Further clarification of the interaction between ferric ions and alginate may lead to better understanding of the properties of Fe-alginate affecting cell culture.

It has been reported that high-M Ca-alginate shows no further changes in material properties after 7 days of exposure to NaCl [20]. In the present study, high-M Fe-alginate gels immersed in culture solution for 8 days also showed little change in gel conditions, such as swelling or polymer content (Figure 4). On high-M alginate gels under the same conditions, NHDF were able to proliferate at the same level as that on high-G alginate gels (Figure 5). These observations suggest that variations in gel condition are detrimental to cell growth.

There is some question as to whether cell growth on high-M alginate films is inhibited by elution of substances from the gels. However, no harmful effects on cell growth were evident when cells were cultured on the surface of tissue culture plates in medium together with high-M alginate gel film (data not shown), thus suggesting that the eluted materials did not influence cell growth appreciably.

Reduction of the concentration of ferric ions cross-linked with alginate did not affect the growth of NHDF seeded on both types of alginate gel (Figure 6). In fact, cells proliferated well on highly swollen high-G alginate with a decreased amount of cross-linked ferric ions. Thus, swelling of Fe-alginate gels was not directly linked to scanty growth of cells cultured on gel surfaces. In the case of high-M alginate, gel conditions were changed during immersion in culture medium, even though the concentration of cross-linked ferric ions was low. It is considered that the percentage of firm cross-linkage in high-M alginate gels is unrelated to the amount of cross-linked ferric ions. In the case of high-M alginate gels under physiological conditions, strong cross-linkage may be maintained, while weak cross-linkage may occur as a result of exchange of cross-linked ferric ions for other competing ions.

## 5. Conclusions

In this study, we have revealed for the first time the effects of Fe-alginate composition on the properties of the gels and their suitability as a culture substrate for cells. Fe-alginate gel with a high G content showed stable cross-linkage with ferric ions, and supported good cell proliferation. In the case of high-M alginate, although partial weak cross-linkage was observed during culture and the cell growth rate was suboptimal, the effectiveness of the gel for supporting cell growth was recognized after it had been stabilized by immersion in culture medium for an appropriate period. In fact, the protein adsorption abilities of the two types of Fe-alginate gels ware similar. Therefore, it is considered that the major factor influencing cell growth on two types of Fe-alginate films is the stability of the gel during culture, and that the mode of cross-linkage between ferric ions and alginate differs according to alginate composition. These results suggest that the composition of Fe-alginate gels could be altered according to their intended purpose. It is anticipated that our findings will be useful for extending the application of Fe-alginate to diverse biomedical fields.
